# Antiseptic Surgery

**Published:** 1891-03-07

**Authors:** 


### ANTISEPTIC SURGERY.

In our last issue we spoke of some of the adverse criticisms that have been passed upon the practice of Antiseptic Surgery, and pointed out in what way their conclusions seemed to us to be unwarranted. In this number we propose to discuss some of the cases in which this method has been of most signal benefit, and the precautions to be used if such results are to be the rule in general practice. We have called this paper Antiseptic Surgery; but in truth this method consists of two very distinct branches —the Aseptic and the Antiseptic—and the two must not be confounded. In *Aseptic* surgery the surgeon is entirely responsible for the progress of the wound. Its essence is to prevent putrefactive fermentation taking place in it. The means used to obtain this result are immaterial. Healing by first intention with the minimum of inflammatory reaction is the type of a perfect aseptic course, but this cannot always be obtained from the nature of the operation, yet a true aseptic course may still be run, and safety to the patient be assured. The *Antiseptic* treatment proper stands in a different relation to the surgeon. Here germs have already had access (or the possibility of access) to the wound, and the surgeon has to destroy them altogether, or so far that the vitality of the tissues can cope with the rest. In this he may or may not be successful, and the latter from no fault of his, but once he has succeeded in his attempt, the future course of the wound is as much in his own responsible keeping as in an aseptic case. At the same time, while admitting that the surgeon may fail in his endeavours from the very nature of the case, it cannot be too constantly borne in mind that in the majority of cases this attempt ought to be successful. The history of compound fractures treated antiseptically amply bears out this statement. Several points call for discussion as to the proper way of carrying out this treatment in these cases. If successfully and thoroughly persevered in it reduces a compound to the condition of a *simple* fracture. This is no mere boast, for it is an established fact that a compound fracture in which there are large fragments of bone detached will unite just as a simple comminuted fracture will without any necrosis, if the antiseptic treatment is properly carried out. That so perfect a result is not oftener obtained is largely due to two causes: i.—Want of free drainage. ii. —Want of persistence in efficient dressings throughout the case.

i. Nothing is more important in such cases than free exits for the copious discharge which must follow any thorough disinfection of the wound. Failure is often the result of hesitating to make free counter-openings in uninjured parts of the limb, and in some cases from not even enlarging openings which already exist. Drainage cannot be too free in the early part of the treatment.

ii. More imperfect results, we suppose, are due to this cause than to any other. The surgeon has probably succeeded in making the wound aseptic—the discharge, however, from the granulation tissue is perhaps free, very likely from the irritation of the dressing; or the granulating wound is simply a little long of healing, and his patience fails; he thinks the wound is quite superficial, and antiseptic treatment no longer necessary, and orders a simple ointment instead. The discharge, which was quite "sweet," becomes sour and irritating—the apparently superficial wound is found to communicate with the fracture, the fermentative process spreads down, and the bone bathed in unhealthy pus must partially necrose.

The result of this is that instead of healing the wound continues to discharge for many weary months, till the necrosed bone comes away; or if this has happened earlier in the course of treatment union does not take place, discharge is profuse, and finally in many cases amputation is the only resource. It must not, however, be supposed that this treatment is never followed by necrosis; this may happen in a case that is running a perfectly aseptic course and be inevitable, but the less the wound is irritated by the antiseptic solutions or dressings employed, the less frequent will be this complication. When once asepticism has been obtained, no more irritation should be produced by the dressings, and the perfectness of the result will depend on the skill shown in this.

In close connection with these cases must be mentioned psoas abscesses, and any abscess connected with a bone, such, for example, as occurs in acute necrosis. In no class of cases does the result of this treatment show more unmistakably than in psoas abscess of vertebral origin. Whereas before they were the despair of every surgeon, they now, in a large majority of cases, admit of complete cure if opened aseptically. But to obtain such a result two precautions are necessary: to open the abscess where antiseptic dressings can be efficiently applied; and never, on any provocation, to leave off these dressings till the sinus is completely and soundly healed. It may be many months before this happens, but, if kept aseptic, it will in most cases close in time, and unlimited care and patience must be exercised. As to the best places for opening the abscess, above Poupart's ligament, or in the loin in some cases, are much safer sites than in the thigh. Even if the abscess point here, it will drain quite readily through the higher opening, and the risk of septic infection (especially in the case of children) is much lessened. We have said nothing about rest in these cases, but obviously that is as imperative as ever. If, therefore, with such possibilities of good success any laxness of treatment allows a psoas sinus to become septic, a very grave responsibility must rest with the surgeon. If efficient aseptic treatment cannot be carried out, it would seem far better to leave the abscess untouched as long as possible, enjoining absolute rest and the usual tonic treatment; and if it begin to point, and some interference becomes absolutely necessary, to aspirate with great caution and perhaps inject (as recommended by Billroth) a solution of iodoform in glycerine (1 in 10), from 1 to 3 oz., or less, being the quantity used according to circumstances. Once more, then, we repeat—in all *bone cases* never leave off antiseptic dressings till the skin wound is quite healed, and the same rule should be made in all joint cases and in operations connected with sheaths of tendons, more especially in the hand.

We have purposely said nothing about the form of antiseptic dressing to be used, for it is not a constant factor. It need only be said that ordinary modesty would suggest that Lister's own materials are *à priori* most likely to be the best, and Dr. White, of Pennsylvania, in a recent valuable paper, has given his experience of the new cyanide gauze in 195 cases, which quite bears out Lister's own testimony in its favour. But the old carbolic dressings are still available and largely used, and with care will be found, in the majority of cases, to answer all purposes. We hear much of the wearisome details of antiseptic treatment, but anyone who has grasped the principles of the method will find all such difficulty vanish, and have no room for any feeling but one of thankful satisfaction.

